# Rights as Relationships: Collaborating with Faith Healers in Community Mental Health in Ghana

**DOI:** 10.1007/s11013-019-09648-3

**Published:** 2019-11-15

**Authors:** Ursula M. Read

**Affiliations:** Department of Global Health and Social Medicine, Kings College London, London, UK

**Keywords:** Traditional and faith healing, Community mental health, Collaboration, Human rights, Ghana

## Abstract

This paper explores the ways in which mental health workers think through the ethics of working with traditional and faith healers in Ghana. Despite reforms along the lines advocated by global mental health, including rights-based legislation and the expansion of community-based mental health care, such healers remain popular resources for treatment and mechanical restraint and other forms of coercion commonplace. As recommended in global mental health policy, mental health workers are urged to form collaborations with healers to prevent human rights abuses and promote psychiatric alternatives for treatment. However, precisely how such collaborations might be established is seldom described. This paper draws on ethnographic research to investigate how mental health workers approach working with healers and the moral imagination which informs their relationship. Through an analysis of trainee mental health workers’ encounters with a Prophet and his patients, the paper reveals how mental health workers attempt to negotiate the tensions between their professional duty of care, their Christian faith, and the authority of healers. I argue that, rather than enforcing legal prohibitions, mental health workers seek to avoid confrontation and manouver within existing hierarchies, thereby preserving sentiments of obligation and reciprocity within a shared moral landscape and established forms of sociality.

## The Teachable Moment: ‘It’s Part of Your Job to Engage with These People’

In December 2016 I accompanied a group of trainee community mental health workers and their tutor, Jacob^1^, to visit the church of a local Prophet in the rural market town of Kintampo, Ghana. The outing was organised as a pedagogical exercise to instruct the students in the ‘sensitization’ of traditional and faith healers,^2^ many of whom treat people with mental illness. Mental health workers are expected to collaborate with such healers, and sensitization is a key aspect of public health work which combines community engagement efforts with education on the signs and symptoms of disease and where to seek treatment. The students were enrolled at Kintampo College of Health and Wellbeing, a leading national centre for the training of public health workers, which in 2011 introduced courses in mental health. Under the midday sun, a large group of around 60 students dressed in the khaki and white uniforms of public health practitioners, set out on the short walk from the college. The Prophet was waiting for us in the shade of a mango tree adjacent to his church. His pastor sat beside him, a large black leather-bound Bible open in his lap. After the exchange of greetings and a prayer, the students were invited to visit the Prophet’s patients.^3^ As we walked towards the grove of teak trees behind the church two men came into sight. Each was shackled at the ankles with his legs either side of a tree trunk. One lay semi-naked in the dust, seemingly indifferent to the stares of the students clustered around him, despite his exposed genitals. The other sat with his hands clasped around the tree trunk, and as the students approached, he began to audibly protest against his treatment, appealing for food. Many of the students were visibly appalled. They gasped, tutted and made shocked exclamations. An improvised shelter of branches and tarpaulin hid a third patient, and some students squatted down to peer at the man inside, who was also shackled to a tree. “This is serious!” said one student, as his colleagues recoiled from the heat and stench. The students gathered around the second man. He told them he had not eaten for several days, as he had been ordered to fast. On his back a number of dark lines suggested he had been beaten.^4^ Jacob addressed the students: “If you do community mental health, these are the things you are going to have to deal with. It’s part of your job to engage with these people”.

The treatment of persons with mental illness in Ghana has come under increasing international scrutiny following the publication of damning reports by influential actors including Human Rights Watch (HRW) and the UN Special Rapporteur on Torture, as well as critical media coverage.^5^ These have exposed the maltreatment of people with mental illness by Christian pastors and traditional healers, in particular the use of chains and enforced fasting (CHRI 2008; Edwards 2014; Human Rights Watch 2012; United Nations 2014). As in other settings, the persistence of such practices has been attributed to stigma, traditional and religious beliefs around severe mental illness and the limited availability of modern psychiatric treatment (Ae-Ngibise, et al. 2010; Atiemo 2014). As Jacob describes, collaboration between mental health workers and traditional and faith healers is advocated as a key strategy to prevent such abuses (Arias et al. 2016; Osafo 2016). The students were being trained as a new cadre of mental health worker, the ‘Community Mental Health Officer’ (CMHO), as part of efforts to ‘scale up’ community-based mental health care in Ghana (Akpalu et al. 2010). The imagined trajectory is that as access to psychiatry increases, inhumane practices, such as the use of shackles and chains, will be replaced by pharmaceutical treatment. As public health workers with skills in community engagement, disease surveillance and health promotion, after their training the students were expected to return to their posts in towns and villages and provide screening and referral for persons with suspected mental illness. Engaging with healers such as the Prophet is envisaged as an important part of their role. The training curriculum states that CMHOs will “liaise with existing providers of mental health care (including ‘traditional’ and other providers) to create working partnerships for the benefit of clients, their families and those who care for them.” (p. 47). According to a recent article which surveyed CMHOs already in post, over half stated that they collaborated with traditional healers (Agyapong et al. 2015).

The scenario at the Prophet’s church was particularly striking to me since the numbers of trainee mental health workers gathered around the shackled patients was visible evidence of the changes that had occurred since I began anthropological research on family experiences of psychosis in Kintampo 10 years earlier. At that time there were no mental health workers in town and it was a long day’s journey to the psychiatric hospitals in the south. The Prophet had gained a significant reputation as someone who could heal madness and consequently became one of my key informants. Over 2 years of fieldwork I witnessed a steady stream of men and occasionally women brought to him for help, many of whom stayed for weeks, months, even years. As with the men encountered by the students, many of these patients were chained to trees on the church grounds and deprived of food for up to 3 weeks at a time. By 2016, in contrast, the mental health workforce in the region had dramatically expanded. There were at least ten mental health workers in Kintampo town itself, plus several others in the surrounding villages. In addition to training community mental health workers, in 2012 Ghana had finally passed a new Mental Health Act after several years of protracted negotiations. The Act explicitly prohibits the involuntary detention of persons with mental illness by ‘unorthodox’ mental health providers such as the Prophet and threatens prosecution for those who violate the law, including imprisonment. Nonetheless, the Prophet’s methods appeared unchanged and his authority and popularity undimmed. What was more, as the most renowned Christian healer for mental illness in the area, the Prophet had become increasingly integrated into local and regional activities as a key ‘stakeholder’ in mental health, widening exposure to his work. He was invited to meetings, forums and focus groups convened by international researchers, NGOs and mental health educators and attended by, among others, health administrators and human rights workers. A letter inviting him to a regional meeting he had attended funded by the UK Department for International Development (DFID) outlined the intention to educate him on “stigma, discrimination and prejudice against persons suffering from mental illness” and “sensitize you on the need to work with Mental Health Practitioners”.

This paper explores this apparent contradiction between the expansion of rights-based mental health policies and activities in Ghana, as part of the broader field of global mental health, and the continued and open use of practices which have been condemned as human rights abuses, contravening both international and national law. I examine the intersections between globalized notions of human rights and collaboration with traditional and faith healers as articulated within global mental health and the ethical reasoning of mental health workers on the ground. To do this I trace the contours of the ensuing debate between the Prophet and the students, as well as analysis of observations, conversations and interviews with mental health workers, to reveal the moral imagination which animated their response. This paper draws primarily on 5 months of anthropological fieldwork in Ghana in 2016, but also on earlier periods ranging from 6 weeks to 20 months between 2005–2008 and more recently in 2017–2018. Fieldwork consisted of observations in family homes, healing shrines and churches and with community mental health workers in and around Kintampo in the Brong Ahafo region, as well as visits to various mental health-related services, organisations and events in Accra, the capital. Conversations, interviews and focus group discussions were conducted with persons who had been diagnosed with mental illness, family members, members of the public, healers, health workers and other relevant actors, such as workers for NGOs and humanitarian organisations.^6^

When Jacob enjoins his students that ‘It’s part of your job to engage with these people’ he means more than a requirement to simply educate healers on the medical approach to mental illness. Rather he points to their responsibility to nurture relationships which facilitate negotiation around the ethics of collaboration and how it could and should be practiced. Like the Tanzanian health workers described by Stacey Langwick, it is Ghanaian mental health workers who must perform the difficult labour of mediating between traditional and ‘modern’ medicine and thereby bring into being new configurations of care (Langwick 2008, p. 431). The meeting between the students, the Prophet and his patients could thus be viewed as a “generative moment”, that is a moment of crisis which exerts “a potentiality which reaches beyond the situation from which it emerges” (Kapferer 2005, p. 103). Such crises stimulate reflection and argument on the ethical course of action (Englund 2008; Mattingly 2014) and thus “do more than illustrate principles at work—they indicate new dynamics in formation” (Kapferer 2005, p. 103). In his concept of ethical assemblage Zigon similarly argues that morality is not a unified totality, but rather emergent in particular social contexts combining “various institutional, public, and personal moral discourses and ethical practices” (Zigon 2010, p. 5). These various strands form the “moral imagination” (Englund 2011, pp. 227–228)—in responding to the suffering of the patients and the authority of the Prophet the good is not predetermined but “must be imaginatively conceived, not simply perceived” (Robbins 2013, p. 457). Indeed, the visit is explicitly designed as a ‘teachable moment’ to provoke ethical reflection and inform students’ future action. It confronts the abstractions of the classroom with the messy realities of practice and brings students face to face with aspects of Ghanaian society that most have until then witnessed primarily at a distance, perhaps in the media or in the reports of human rights organisations, and in which different actors have stakes—healers, health workers, the family, and, not least, the patient. The shock and disgust, even shame, deliberately engineered through this close encounter, forces students to reflect on the ethics of working with such healers. However, whilst human rights abuses by traditional and faith healers are often portrayed as a uniquely cultural challenge for Africa and other low-income settings (Hughes 2018), the situation points beyond this to ethical tensions in approaches to the care and control of people with mental illness in all societies, regardless of resources.

## Human Rights and Social Obligations

For the students, what rendered the interaction ethically uncertain were the ambiguous lines of authority and responsibility between health workers, the healer as a professed ‘man of God’, and the families as ‘therapy managers’ (Janzen 1987). In negotiating the terms of their relationship with the Prophet mental health workers are required to navigate potentially conflicting ethical and moral considerations. As health professionals and citizens of one of Africa’s most celebrated democracies, the students are familiar with professional ethics and ideals of citizens’ and patients’ rights. Some claimed that their recognition of people with mental illness as fellow ‘citizens’ was what had inspired them to train to work with people who in wider society are highly stigmatised. They could recite with facility the five principles of medical ethics: respect, justice, autonomy, beneficence and do no harm. As one student put it: ‘They guide us in our work. So we don’t cause harm to the patient’. At the same time, the majority of the students shared the Prophet’s faith, recognising the potential of spiritual powers to cause sickness and bring about divine healing. Christianity, particularly Pentecostalism, deeply pervades public life and moral discourse in Ghana (Meyer 2011). Religious teachings may be viewed as enshrining a higher order morality which supersedes professional ethics or even national laws, particularly where there is widespread mistrust of political authority and fears of corruption (Okyerefo 2011). Furthermore, there are inherent tensions within the Mental Health Act which outlaws the coercive practices of healers whilst urging mental health workers to collaborate with them. Despite moves to sensitize and educate healers such as the Prophet, questions remain as to how in practice mental health workers might negotiate the kind of relationships through which to meet these conflicting imperatives to regulate and collaborate.

Didier Fassin describes how ‘humanitarian reason’, evoking a moral imperative to relieve human suffering, has come to justify government interventions from military action to aid (Fassin 2012). Such suffering is often portrayed as arising from a particular moment of crisis which provokes an emotional response—outrage, compassion, alarm—and a sense of urgency (Calhoun 2008). In this humanitarian imaginary change is often conceived as coming about not through a slow process of negotiation, but through moments of dramatic and liberating rupture. In respect of relieving the suffering of persons with mental illness in Ghana, such rupture may be conceived as breaking relationships with seemingly callous and misguided healers as much as a ‘breaking of the chains’. Such was the tone of a 2017 media article reporting how the Mental Health Authority, set up to oversee mental health care in Ghana, had “stormed” a prayer camp and “released 16 mentally challenged people from chains”.^7^ In this approach, legislation is promoted as a vital tool to protect the human rights of the victims through sanction and punishment of the perpetrators. However, Harri Englund (2011) argues in his analysis of human rights in Malawi that such ruptures risk disrupting vital networks through which claims for support or collaboration may be met, particularly in low-income settings with fragile health and social welfare systems. Englund argues for a move away from the notion of rights as primarily a legal construct, in which ideals of parity erase hierarchies, to consider rights as constituted through particular relationships and the obligations they entail (Englund 2011, p. 228). Human rights gain traction not through what they are in the abstract, in formalized conventions and laws, but through the ways in which they articulate with existing modes of sociality, such as rank and status (Selby 2012). Where policy describes an undifferentiated category of traditional and faith healers who may be castigated as abusers or promoted as potential collaborators (Cooper 2016; Nichols-Belo 2018), mental health workers must work face-to-face with specific individuals who participate, often as respected authorities, within the same community. The obligations which inhere in such authority may be those which enable change to occur without a radical transformation of existing hierarchies. In this way rights are emergent within the situated contingencies of the present, rather than deferred to a utopian future (Englund 2011, p. 15). This helps us to consider the incremental ways in which change may be pursued precisely through maintaining the primacy of relationships and their constitutive obligations (Englund 2011, p. 224).

## The Treatment Gap, Human Rights and Traditional and Faith Healing

Traditional healers have long been advocated as important resources for mental health care in Africa, providing services which chime with ‘traditional belief systems’ (Twumasi 1979). Indeed, much early work in transcultural psychiatry was concerned with exploring the efficacy of traditional healing and how, in the absence of formal mental health services, psychiatrists and traditional healers might work together. In this literature traditional healing was commonly depicted as a form of African psychotherapy (Field 1955; Forster 1962; Prince 1964), addressing the social and psychological effects of mental disorders.^8^ Traditional healers were often rather loosely defined, but generally described as drawing on long-held practices which perceived mental illness as arising from supernatural or spiritual causes.^9^ The World Health Organization (WHO) lent support to this approach in the declaration of Alma Ata which advocated collaboration with traditional healers as part of efforts to expand access to primary health care. Such calls were renewed in the 1980s following the devastating effects of structural adjustment on African health systems and human resources for health (Bannerman 1983). Since then varieties of Christian healing have hugely expanded in many sub-Saharan African countries and in some cases may exceed the popularity of traditional practitioners. In Ghana, for example, residential ‘prayer camps’ have proliferated since the 1990s offering a diversity of practices under the umbrella of Pentecostal or Charismatic theology (Asamoah, et al. 2014). The potential of traditional and faith healers as ‘a key player in the mental health care system’ (Patel 2011) is widely promoted within global mental health, particularly in view of the so-called “treatment gap”, that is the difference between the estimated burden of mental disorders and the availability of mental health services (Kohn, et al. 2004). A recent review in the Lancet suggested that “In areas where formal psychiatric services are scarce or unaffordable, traditional healers […] provide a potentially valuable source of mental health care” (Nortje, et al. 2016, p. 154). Perceived advantages include accessibility, cultural acceptability, a holistic approach and less stigma (Gureje, et al. 2015, p. 173). Gureje and colleagues describe collaboration as relationships in which ‘traditional or conventional medicine practitioners remain autonomous and independent but co-operate fully; for example by referring patients to each other, or consulting on complex cases’ (Gureje et al. 2015, p. 173). In many projects within global mental health, as with the Prophet, traditional and faith healers are seen as important stakeholders to be consulted and sensitized in the development of community interventions (Alem et al. 2008). This approach contributes to a conscious cultural adaptation of global mental health interventions, countering critiques of global mental health as simply cultural imperialism (Summerfield 2008).

Despite such calls for collaboration, there is also widespread concern regarding human rights abuses by traditional and faith healers. The circulation of emotive images of men, women and children in chains, as featured in the Human Rights Watch report (Human Rights Watch 2012) and an article outlining research priorities for global mental health in the leading science journal *Nature* (Collins et al. 2011), provoke international outrage and calls for urgent action. Framed in the globalised language of human rights violations such appeals tap into older colonial discourses of the global south as primitive and savage, in need of rescue by more enlightened regimes (Mutua 2001). In keeping with this modernising narrative, legislation and international conventions are envisaged as key tools to protect the human rights of persons with mental illness and bring those who contravene these rights to justice. The UN Convention on the Rights of Persons with Disabilities (CRPD), which Ghana ratified in 2012, brings mental health under the umbrella of disability and, in keeping with other rights-based declarations, compels signatories to take action to prevent persons with disabilities “from being subjected to torture or cruel, inhuman or degrading treatment or punishment’ as well as ‘exploitation, violence and abuse” (United Nations 2006). The reform of mental health legislation and policy in line with the CRPD forms a central strategy in the current WHO Mental Health Action Plan to protect human rights within psychiatric services as well as traditional and faith healers (WHO 2013).

Over the last decade Ghana has become increasingly drawn into the networks of global mental health within Africa. Compared to other countries in the region, Ghana benefits from a stable economic climate and governance, as well as a comparatively well-established mental health system. These factors have attracted researchers, NGOs and international donors to launch various mental health initiatives which have increased over the last decade as international funding and interest has grown. The deployment of CMHOs resulted from a partnership between the college, the Ghanaian Ministry of Health and a mental health trust in the United Kingdom’s National Health Service^10^ and represents a form of ‘task-shifting’ in which health workers with lower levels of training are deployed to address shortages in specialist personnel (Petersen et al. 2011). Such processes form central logics within the vision of global mental health with the aim to expand access to cost effective psychiatric treatment in low- and middle-income countries (Lancet Global Mental Health Group 2007). Many of these initiatives are shaped by an explicit rights-based approach, with talk of the need to tackle stigma and human rights violations through participation, empowerment, and advocacy. Members of WHO’s mental health division were closely involved in drafting the Mental Health Act, providing consultation, technical assistance and training. WHO praised the Act as “a very progressive mental health law” and cited it as an example of ‘best practice’ for other member states (WHO 2007). The Act formalises calls for collaboration with traditional and faith healers, which it terms “providers of unorthodox mental health care”, whilst promoting “the best interests of persons with mental disorders” through mandating state mechanisms of oversight, including a visiting committee to inspect their facilities. The Mental Health Act is often described by activists and policy makers in terms which promise a utopian future for mental health services in the country. At a press conference for World Mental Health Day in October 2016, for example, the CEO of the Mental Health Authority in Ghana claimed that implementation of the Act would “build mental health in Ghana to a world class standard” and there would be “no more chaining”.

Despite these innovations, coercive practices such as chaining and deprivation of food continue, as I observed in Kintampo and elsewhere. Mental health leaders in the country attribute this in the most part to significant delays in implementing the Mental Health Act. Specific legislative instruments to enact the law have not been passed, government funding has yet to materialize and the visiting committee has not been established. Consequently, regulation and oversight of traditional and faith healers remains piecemeal. Limited resources and weak infrastructure also undoubtedly constrain the ability of mental health workers to engage with healers. The lack of ringfenced funding alongside wider health system constraints mean that mental health workers struggle to access the resources they need to fulfil their role, including supplies of psychopharmaceuticals (Oppong et al. 2016) and transport for community visits. However, in the encounter between the students and the Prophet it was evident that change was neither predicated solely on full implementation of the Mental Health Act nor on the availability of resources. At the time the necessary pharmaceuticals were available and there were actors, including the police and human rights bodies, such as the local office of the Commission on Human Rights and Administrative Justice, who could in theory be called on to enforce the Act and protect the rights of the Prophet’s patients. Yet despite their evident shock at his methods, the discussion which ensued uncovered a rather different logic through which the students appraised the Prophet’s practice and which shaped their approach to collaboration.

## ‘Can You Back that Up with Scripture?’: Thinking with Theology in Mental Health

After visiting his patients the students were invited to ask questions of the Prophet. The students began with questions about the success of his treatment—“Had anyone been healed?”—and the practical implications for care—“What happens if the person needs to use the toilet or is on her menses?” But the discussion quickly turned to the theological rationale for the Prophet’s methods. A student asked, “How do you identify if a condition is spiritual or physical?” The Prophet repeated what he had told me years before: “God did not create human beings to get sick. Whatever sickness you get has a spiritual cause”. He gave a citation, “Mark chapter 9, verse 17”, and his pastor opened the Bible. At the same time several students looked up the verses on a Bible app on their mobile phones. As the pastor read the Bible aloud the students listened attentively, some followed the words on the app. The verses told the story of man who brings his son to Jesus and tells him he is possessed by a spirit which, “wheresoever he taketh him, he teareth him: and he foameth, and gnasheth with his teeth, and pineth away” (Anon 1996, p. 788)—a picture which clearly resonated with students who had witnessed epileptic seizures.^11^ After he has cast out the spirit, the disciples ask Jesus why they could not. Jesus replies: “This kind can come forth by nothing, but by prayer and fasting.” (ibid.)^12^ The Prophet repeated this final phrase for emphasis: “The sickness will not go except through fasting and prayer”. Several students nodded in agreement.

Some, however, were not satisfied with this explanation. “Does the patient or the pastor do the fasting?”, one student asked. The Prophet replied that the patient does the fasting, but he fasts at the same time. He compared it to hospital treatment: it is the patient who must take the medication, not the doctor. A male student interjected: “But the purpose of fasting is to commit yourself to God”. Jacob interrupted jokingly: “Can you back that up with Scripture?” There was some laughter, but the student continued earnestly: “Looking at their condition, if they are subjected to fasting, I don’t think they can pray.” Jacob clarified his point: “You are asking if their mental state is not good, can they know what they are doing?” The Prophet responded: “They don’t know they are fasting at first, but when they feel hungry they realise what they are doing”. Jacob tried to settle the issue: “He’s done the work for 31 years. Once you are sick, you are supposed to be fasting and praying”.

As this exchange illustrates, in contrast to national and global discourse, and despite a familiarity with notions of rights, the students’ ethical questioning was framed through theological debate around the Biblical foundations for the Prophet’s practices and his claims to spiritual authority, rather than in terms of human rights and their possible violation. The students tested the Prophet’s skills in Biblical exegesis, seeking to determine whether he was truly a ‘man of God’.^13^ Rather than closing down discussion by asserting inviolable rules, such enquiries open spaces for negotiation and set out a shared moral terrain on which collaboration could take place. For the students, as for many Christians worldwide, the Bible is not a fossilised moral code, but an indispensable guide to everyday life whose ambiguous content requires careful reflective interpretation, often with the aid of a more learned or respected fellow believer, such as the Prophet (Engelke 2004). The students take seriously the possibility of Biblical foundations for practices which may contravene national laws. As one student later told me, “We are talking spiritual that he was basing on. He was saying that this kind of problem or condition needs fasting and praying. That’s what he is basing on. That is why he has been letting them go through that fasting.” In this encounter the ranked relationship between the Prophet and the students is crucial. As Jacob’s comment reveals, the Prophet is an older man who commands respect among many families and has longer experience of dealing with mental illness than any of those who visit him. Hence his opinions cannot simply be dismissed, however harsh or shocking his methods. What is more, whilst the chains are a highly visible and disturbing aspect of his treatment, he also provides shelter, support, and in some cases food for people who are commonly ignored and stigmatised in wider society. One grandmother whose granddaughter I had observed chained and fasting at the Prophet’s church described how seeing her granddaughter in chains in the rain had moved her to tears. Nonetheless she expressed faith in the efficacy of the fasting and prayers and explained how when the fasting period was over the Prophet would provide yam and a small cash donation so that she could feed her granddaughter. As she told us: “the fasting we did and the prayers made her well. I would never be ungrateful to the Prophet. He’s done very well.” Similarly, another mother expressed gratitude to the Prophet who through his methods had prevented her son from going to smoke cannabis which she believed contributed to his illness and had asked for no money in return.

Nonetheless the students’ questions reveal that the Prophet’s moral authority cannot be taken for granted by virtue of his status or even his philanthropy. Pastors may perform miraculous acts, even heal sickness, but one cannot be certain whether their power comes from God or the Devil (Read 2016; Shipley 2009). There is a lively critique in Ghana of ‘false prophets’ who have little theological training and exploit their congregations for material gain (Daswani 2013; Shipley 2009)^14^. Questions around theological interpretation become further complicated when combined with uncertainty regarding the capacity of persons with severe mental illness to make rational decisions. Fasting is widely practiced among Christians in Ghana, usually accompanied by vigorous prayer and undertaken when seeking spiritual guidance or making requests, for example for healing or business success. Thus the student’s concern as to how people who had psychosis, or madness, a condition in which the person is considered to be “not in his right sense”, could engage in the kind of conscious prayer the practice demanded. As another student later asserted, such lack of awareness rendered the exercise starvation rather than fasting: “He forced the person to not eat. It’s not fasting, it’s starving. For the people who are doing it, they don’t even know what they are doing. It’s the man that is compelling them to do it….” One of Jacob’s colleagues expressed a similar view. He argued that the Prophet misinterpreted the Bible—it was he who should fast and pray and not the patients. Such diversity of opinion nonetheless leaves intact the potential for divine intervention and made it difficult for mental health workers to dismiss the Prophet’s claims entirely. It was this potential which animated the next phase of the discussion.

## “We Have What the Doctors Do and We Have What the Pastors Do”: the Power to See

Whilst the Bible is respected as the divinely inspired ‘word of God’, spiritual authority is judged not through skills in textual exegesis alone, but also through evidence of spiritual perception, commonly referred to in Ghana as having ‘four eyes’. Only those who can ‘see’ can divine the ultimate cause of illness.^15^ This spiritual vision cannot of course be interrogated in the same way as Biblical knowledge or subjected to similar forms of reasoning, and thus introduces a deeper layer of ethical uncertainty. It is widely accepted that not everyone who calls himself a pastor or prophet can ‘see’, even though they may claim to do so. But unless one has ‘eyes’ oneself, one can never be certain. As one student put it, “You don’t know whether they have the eyes, they saw, or they are also just saying it.” In the debate with the Prophet, one student dared to broach this tricky subject: “You said you have a belief that it’s been caused by spiritual factors. What do you see in the spiritual realm?” His question asked the Prophet, in effect, to prove his spiritual powers. As he acknowledged, this came close to breaching decorum since it posed a direct challenge to the Prophet’s claims to spiritual authority. The pastor responded in terms which aimed to close down further questioning: “You can’t show spiritual matters to those who do not have spiritual sight”. The student persisted: “Do you see things coming out of the patient for example?” Another student intervened in an attempt to clarify the issue. As she began Jacob teased her, “Can you also see?”, but she continued: “There are some things God gives you the go ahead to say, others he doesn’t. If it is an aunt or uncle who bought the illness you can revoke that spiritual illness. But others God will not give you the go-ahead to say it”. The student referenced a popular perception of a means by which illness might be spiritually given to someone—*nto yaree,* a ‘bought illness’, is one where an illness might be purchased at a shrine to inflict on an enemy, usually a family member. ‘Revoking’ the illness referenced the ways in which pastors and believers attempt through prayer and other rituals to undue the effects of such malign spiritual actions. As the student described, sometimes the assumed perpetrator is named, but often the accusation is left open to interpretation, since naming the perpetrator risks “creating enmity”, as another student later put it.

Like the Prophet, many if not most students and mental health practitioners are convinced that at least some mental illnesses are spiritual, caused by the malign actions of another human being such as a curse, witchcraft or the use of ‘bad medicines’. Yet without the spiritual vision to ‘see’ them for health workers such issues layer additional uncertainty on top of the already puzzling trajectory of mental illness. Arthur, a psychiatric nurse explained:

I can’t see them. Because you know, there have been situations where somebody suddenly exhibits mental, er, abnormal behaviours and then they go to a spiritualist or a pastor or a traditional healing place, and they do the prayers, or they use some herbs to bath the person, or give the person some concoctions or something, and then immediately it goes off [away]. And for years the person hasn’t exhibited any symptoms again. And at the same time the person’s also not on any form of medication. […] So, in a way it tells us there may be spiritual causes.

However, health workers evade voicing suspicions of spiritual illness since to do so implies the actions of a human agent and carries potentially disruptive consequences. As one student said: “You can’t tell your client or your patient that it’s caused by spiritual because telling them can lead to them harming or doing something bad to their fellow human being”. Furthermore, another pointed out, naming a spiritual cause could prevent the person from receiving psychiatric treatment, thereby transgressing the professional boundaries of the mental health worker’s role: “As a CMHO in that situation you will just have to do your professional work, educate the family. You don’t let them know that it is a spiritual cause. Because when you tell them it is caused by a spirit, then they will not comply to your treatment.” Pentecostal pastors on the other hand display no such equivocation. They claim the power to definitively discern spiritually inflicted sicknesses and to undo or ‘break’ them through prayer or ‘deliverance’. For Arthur spiritual discernment explicitly informed the lines of authority in negotiations with healers: “People who use spiritual means to treat, they claim they see certain things about the patient which we don’t see. So because we don’t see, we cannot challenge them, we can only advise them. And if they take it, they give us the permission to intervene.” From this perspective the relationship between the health professional and the healer is structured according to a hierarchy in which the healer permits the health professional to treat his patients, rather than the other way around. Unlike the Mental Health Act which views mental health workers as the ultimate authority on mental illness, this acknowledges the limits of the health worker’s powers, and the hierarchy under which the health worker as a believer may defer to the superior insight of a spiritual teacher.

The shared belief in the possibility of spiritual causation to mental illness and their faith in divine healing means that students see little contradiction in addressing both biomedical and spiritual factors and accept a requirement for services such as those offered by the pastor, despite his questionable methods. As another nurse put it, “We have what the doctors do, and we have what the pastors also do”. Thus, collaboration is a two-way process in which professional consultation and referral goes both ways. Pharmaceuticals can control the symptoms, whilst prayer, fasting, confession, or deliverance from evil spirits, curses, or witchcraft address the underlying spiritual cause. Health workers routinely encourage patients to combine “medicine and prayers”. One student told me: “As they take their medication, they should pray alongside, then if it’s spiritual…”. This then covers all possibilities, even where, as was often the case, the cause of the affliction was uncertain (Read 2017). Indeed, whatever treatment one receives, whether from the doctor or the pastor, in the students’ understanding, God is the ultimate healer.^16^

## Calming Madness: Equating Chains and Injections

While prayers are thus valued as an adjunct to medical treatment, and the theological rationale for fasting is open to debate, shackling patients can be located at what Brodwin and Velpry term the “ethical limits” of psychiatric practice (Brodwin and Velpry 2014). However, in general the need for restraint was not questioned, although the particular methods might be deplored. Mental health workers in Ghana appear little troubled by the ethics of involuntary sedation^17^ and more morally concerned with responding to families desperate for help with a relative who is agitated or aggressive, disturbing the household and often the wider community. Such behaviour, widely associated with madness, is what most often what propels families to approach healers or health workers. The enforced administration of injections of powerful tranquilisers to calm aggressive or agitated patients constitutes a major part of mental health workers’ performance of their professional expertise, and the impressive effects of sedating medication in calming disruptive behaviour demonstrate their potential value to the community. As one community psychiatric nurse explained,

when you are called to attend to a case, after you have sedated the client, because the client was aggressive, destructive, and then causing a lot of troubles to the family and the friends over there, when you are called there, and you are able to calm the client, it’s like you start gaining some respect from them.

Here the patient can be understood, in anthropological terms, to be the family and community rather than the afflicted individual.^18^ From this perspective, the sedating properties of psychotropic medication are openly valued.^19^ Psychopharmaceuticals are explicitly presented in Ghana, as in global mental health programmes more generally, as the humane and modern alternative to mechanical restraint. The Ghanaian Mental Health Act thus permits the use of “involuntary seclusion or minimal mechanical restraints”, but “only where there is imminent danger to the patient or others” and “tranquilisation is not appropriate or readily available”. Both mental health workers and the Prophet spoke of their need to calm the patient, by force if necessary, with the goal to return the person to “his right mind”. When a student asked whether the patients were “in chains 24/7”, the Prophet explained that in hospital they give injections to calm the patients; he uses chains for the same ends. Fasting is his method to achieve a cure, and the chains ensures patients fast who would otherwise leave to seek food. When another student asked: “Can you not link with the hospital to give medication instead?”, the Prophet’s response was brusque: “The hospital is there. If you want to go, go.” Jacob added: “He doesn’t ask people to bring their relatives here. If you come here, they know he will put you in chains”. Once the family put their relative in the Prophet’s hands, he becomes the patient’s (and the relative’s) spiritual father, with all the authority and responsibility that inheres in that role. As one father whose son had been treated by the Prophet explained: “If you take your sick person to him, he would say he’s the one in charge of the person’s treatment. He would tell you that this is the method I’m going to use to treat your sick person. You can’t say you won’t allow it. So, whatever he says, you have to abide by it.” Indeed, since the passing of the Mental Health Act, the Prophet has developed a ‘letter of undertaking’ cast in quasi legal language which he asks relatives to sign to signal their consent to his treatment, including fasting. This paper technology mimics the procedure for involuntary hospital commitment under which the family and the psychiatrist assume legal responsibility for decisions around treatment should the patient be judged to lack decision-making capacity.

This perceived need to control dangerous behaviour has become the point of entry for mental health workers in their collaboration with traditional and faith healers. As John, one of the CMHOs, put it: “why not work in collaboration with us so that when somebody comes and you think it’s violence or whatever, we take care of that, and when the person is stable he comes to you for your prayers?” The Prophet and health workers thus equate injections with chains as achieving the same ends by different means. This equivalence enables mental health workers to find common grounds for intervention, recognising chains, in the words of Arthur, as “cruel” but necessary, much as they ethically justify the use of coercion in their own practice. It retains respect for the expertise of both healers and health workers and enables health workers to present a simple argument to replace the chains with “our injections”. As one student said: “if they cooperate with us, and they are able to give them medication that will calm them down, there will not be a need for them to chain them again. So if they collaborate with us it will be ok. They will stop the chaining.” However, this reasoning enables the Prophet in turn to present the two practices as not only functionally but morally equivalent—a family may choose the hospital and its injections or the Prophet and his fasting and chains. In effect, this equivalence justifies rather than challenges the Prophet’s methods.

From the perspective of the Mental Health Act, the Prophet’s chains contravene human rights whilst psychotropics fulfil the right to treatment. The Prophet, mental health workers and the terms of the Mental Health Act however all sidestep the broader human rights concerns regarding the involuntary treatment of persons with mental illness, as well as the perception of people with mental illness as disposed to violence and lacking decision-making capacity. Debates regarding the ethical basis for involuntary treatment have long problematised the application of rights-based approaches to mental health around the globe and have attained new prominence with the passage of the UN CRPD. Advocacy groups have used the convention to support their argument that involuntary treatment of whatever kind represents a violation of human rights. Others, predominantly psychiatrists, argue that withholding treatment in the absence of consent could breach the person’s rights to treatment (Freeman, et al. 2015). This issue remains hotly contested yet such controversies have largely been muted in discussions around improving access to psychotropic medication in low-income countries. Where psychiatry is newly taking hold as modern treatment set against the barbarism of ‘traditional’ methods of restraint such questions may be of even less concern. In promoting collaboration between mental health workers and traditional and faith healers, there is little acknowledgement of the potential irony of transferring persons from one form of coercion to another. This is a real risk in Ghana where the psychiatric hospitals have also been condemned on human rights grounds (Méndez 2014).

## Collaboration or Censure

Despite the fact that the use of chains is illegal whilst involuntary sedation, subject to the conditions of the Mental Health Act, is not, in practice this difference does not structure the relationship between the Prophet and the health workers who seek to collaborate with him. On the one hand, only the Prophet could claim the power to truly heal, as opposed to treating the symptoms. John admitted that his own attempts to collaborate with the Prophet had not been entirely successful: “he’s been saying that it is only the prayers that treat mental illness entirely […] he’s saying that even mental illness it’s not caused by what we think is the main cause, but it’s spiritual. And it is the demons who are living in the person, so the prayers drive the demons away”. The Prophet’s refusal to collaborate on the terms suggested by mental health workers exposes the uncertain lines of authority along which the relationship is negotiated in practice. The potential for collaboration, as Arthur recognised, lay largely in the hands of the healer, rather than, as suggested in the language of the Mental Health Act, in those of the health professional. The offer of help to manage violence is welcomed by some healers, but others, like the Prophet himself, might reject it on the grounds that it questions the efficacy of their methods and the rationale for their use.

In contrast to the confrontational approach suggested by rights-based discourse, in interviews and conversations health workers described how they patiently sought to nurture a relationship which enabled collaboration without challenging the hierarchy between them and the healer. This included deliberately avoiding direct criticism and confrontation, as one psychiatric nurse told me: “You know, when you go to the community and you start to sabotage them, telling them that what they are doing is not good, they will make your work difficult for you […] they’ll be fighting with you, thinking you want to come and take over their work”. Another nurse, Abednego, explained that enforcing the Mental Health Act could in his view create enmity, not only between the health worker and the healer, but the entire community: “when they maltreat a client and you take them to human rights, and let’s say they are charged or they are being detained in the cells or the police station for a while just to punish them for abusing the rights of a client, you wouldn’t be liked”. As Abednego indicated, in Ghana direct confrontation, particularly with those higher up the hierarchy or from whom one wishes to elicit a favourable response, is generally avoided. Creating enemies is potentially dangerous, as they may seek to harm or obstruct you. Importantly mental health workers must strive to maintain their reputation as public servants in a context in which health workers can be suspected to be receiving ‘perks’ or otherwise feathering their own nest at the expense of the public purse. Avoiding enmity and maintaining good relationships is thus vital for mental health workers to be accepted in the community and use their moral capital to work for change. Importantly, such change was perceived to require an investment of time: “You go visit and give them education, you educate them on mental health. So if you are able to do that for some few years they also know something about mental health […] later on they will come in”.

However efforts to establish relationships which enabled collaboration went beyond education. Whilst visiting the Prophet Jacob showed us a large concrete platform which had been constructed among the trees where the patients were chained. He explained that an earlier student cohort had raised money to begin constructing a shelter for the patients. When they graduated, the work was suspended, but he was hoping it would be continued by the new cohort. Our visit to the Prophet concluded with a ‘vote of thanks’ from the class leader and a final prayer from his pastor. The class leader pledged to give ‘any time we have to support the facility and advance the work’. Whilst from the perspective of international agencies this may carry “the odour of collusion” (Hughes 2018, p. 10), from the perspectives of the actors involved such support signifies recognition of the Prophet’s efforts in a shared field of endeavour, responds to his requests for support, and demonstrates investment in the relationship. As Jacob explained to me later, inviting the Prophet to meetings and engaging him in training mental health workers was also a means to show recognition of his authority. In turn, as Englund (2011) argues, this establishes obligations for a reciprocal response by the healer to the requests of the mental health workers. The building of the shelter is thus a material testament to the intention of the Prophet and the health workers to work together for the benefit of persons with mental illness. However, its partial construction and the ongoing chaining of the patients is testament also to the challenges and contradictions in fulfilling that aim.

## Conclusion

In this paper I have illustrated how mental health workers in Ghana seek to establish the ethical grounds on which to collaborate with healers whose practices contravene global and national rights-based directives. In doing so they draw on a shared moral imagination informed by Christian theology and the potential for spiritual causation of mental illness, as well as a perceived necessity to enforce treatment where reason is impaired. Their approach exposes the uncertainties and contradictions within the human rights discourse of global mental health, and the practical and ethical challenges for mental health workers when urged to censure human rights abuses through establishing collaborating relationships with healers. When health workers confront ethical dilemmas within the lived sociality of face-to-face relationships, the ideals of human rights directives too often remain abstract policy rather than guides for practice. As Langwick argues, pragmatism is integral to clinical work: ‘institutionalised forms of integration imagined in health development programs differ epistemologically and ontologically from the more pragmatic relationship between traditional and modern remedies, maladies, healers, and knowledge in the clinic’ (2008, p. 429). For Englund, equality is “an ethos rather than a policy […] an obligation to be realized in particular situations rather than as an ever-elusive goal to be pursued” (11, p. 225). It is in the particularities and uncertainties of situated interactions that relationships are created between healers and health workers, weaving together ways of knowing with established forms of sociality in order to actively figure out what collaboration might mean in practice, and how lines of authority might be negotiated. For mental health workers relationships with healers, families and communities are vital to conduct challenging work in conditions of scarcity. The act of fostering a relationship rather than implementing sanctions seeks to draw forth reciprocal obligations within which both sides can make claims for support, consultation and co-operation. This avoids the disruption of vital networks of support and may open pathways to incremental and meaningful change. Compared to the time-limited theatre of interventions sponsored by international agencies, for mental health workers such relationships endure and shape the social field within which they conduct their work as agents of change within the community.

However, this is not to brush aside the fact that whilst waiting for societal change, individual suffering continues. The very real agonies of being chained and deprived of food cannot be ignored. Over the years I and my research assistants have spoken with many patients in healing churches and shrines who voice their suffering and anger at the treatment they have received. One patient whom I visited regularly in 2008 while he was chained and fasting at the Prophet’s church is now receiving treatment from community mental health workers. When interviewed about his experiences in 2016, he told us: “It is not right because I have not done anything. Even if I have done something, it is not right for him to chain me”. Nonetheless he continues to voluntarily attend the Prophet’s church whilst taking medication, combining medicine and prayers, as the mental health workers advocate. Indeed, several former patients actively participate in the life of the church, leading prayers, reading the Bible, and even helping to chain new patients. More recently I have been told of a few patients at prayer camps who have sought redress either through the Commission on Human Rights and Administrative Justice, set up to mediate in cases where human rights are perceived to have been violated, or through the police. Not all cases were followed through by the complainant and no one seems to have been imprisoned or fined.

The question remains as a whether a focus on collaborations between mental health workers and healers might serve to mask a broader need for adequately-funded health and social services, including family support, which may prove more effective in reducing the use of coercion and improving care. A human rights worker indeed alleged that the government of Ghana was taking an ‘escapist approach’ and ‘institutionalising illegality’ by allowing prayer camps and traditional healers to contain persons with mental illness rather than invest in services. As Fassin observes, evoking moral sentiment through exposing the suffering of others provides a seemingly unquestionable rationale for urgent intervention (Fassin 2012). This humanitarian reason is visible in the appeals of international organisations, including Human Rights Watch, WHO and the United Nations, and of other global mental health actors and is enshrined in the Ghanaian Mental Health Act. However, without the commitment of resources, this reasoning has located the moral responsibility for change in the hands of individuals—families, health workers and healers—leaving untouched the structural inequalities and political indifference which contribute to the neglect and mistreatment of persons with mental illness, many of whom come from the poorest sectors of society. Negotiating rights through relationships may bring about incremental change in particular cases, but without wider state support persons with mental illness and their families face enduring ethical and practical challenges in receiving and providing care.

## Footnotes

1 All names have been changed.

2 ‘Traditional and faith healers’ is the term commonly used in the global health literature to refer to a diversity of non-allopathic healing practices. I use it here as shorthand to refer to the wide and dynamic variety of healers who predominantly operate outside the state health system and draw on indigenous traditions or Christian (also Islamic) cosmologies. In Ghana the Mental Health Act uses the term ‘non-orthodox healers’. However the English term ‘traditional healer’ is widely deployed by health workers and policy makers to refer to healers who draw on indigenous cosmologies and ritual forms. These include the use of herbal medicines and rituals of divination and sacrifice. The term ‘faith healer’ is less used by practitioners or policy makers who are more likely to refer to ‘pastors’ and ‘prophets’. ‘Pastor’ (*ɔsɔfo* in Twi) is used to describe the leader of a Christian church (usually drawing on Pentecostal or Charismatic Christianity). Some of these pastors, as in this paper, describe themselves as prophets (Twi *ɔdiyifo*) which implies particular spiritual insight and healing power. Not all pastors offer healing, though most who call themselves ‘prophets’ do.

3 I use the English term ‘patient’ to refer to those receiving treatment for mental illness from the Prophet. The term used in Twi is *yarefo* which translates as ‘sick person’.

4 The beating may not have been carried out at this church and the scars may have resulted from earlier mistreatment. Beating of persons showing signs of mental illness by members of the public, family or at churches and shrines was commonly reported to us, and we saw evidence of this in scarring and lesions, though we did not observe a beating first hand.

5 A documentary screened on the BBC in 2015 entitled ‘The World’s Worst Place to be Disabled’ depicted persons with disabilities, including mental illness, confined at prayer camps https://www.bbc.co.uk/programmes/b064449w.

6 Fieldwork was funded by the Social and Economic Research Council (2006–2008), the European Research Council as part of the Cermes3 project Globhealth: From International Public Health to Global Health (2016), and the Wellcome Trust as part of a Kings College London collaborative grant on Mental Health and Justice (2017–2018). Research assistance and language support was provided by Awudu Isaaka, Solomon Nyame (Kintampo Health Research Centre), Lionel Sakyi (University of Ghana) and Osei Isaac Kwame (Kwame Nkrumah University of Science and Technology). Ethics approval was obtained from University College London (2005–8), Kintampo Health Research Centre (2006–2008), Ghana Health Service (2016, 2018) and Kings College London (2017–2018).

7 Shirley Asiedu-Addo, *16 Chained Mentally Challenged Rescued at Prayer Camp,* The Daily Graphic 3 July 2017. This was carried out at one of the prayer camps visited by Human Rights Watch in 2012.

8 The efficacy of herbal preparations, in particular rauwolfila, was also investigated (Prince 1960)

9 The term ‘traditional healing’ emerged during the introduction of biomedicine by Christian missionaries and colonial governments. Several writers have highlighted the work of translation and elision which has gone into the construction of this terminology which embraces a huge variety of practices and falsely opposes unchanging tradition to scientific modernity (Pigg 1995). As Langwick (2008, p. 437) describes, the term has, however, gained ‘epistemic and bureaucratic weight’ in postcolonial Africa.

10 For details of the partnership with Southern NHS Trust see http://www.thekintampoproject.org/.

11 Although a neurological condition, epilepsy is historically treated within psychiatric services in West Africa and is now encompassed by the term ‘mental, neurological and substance use disorders’ (MNS) deployed in the Global Burden of Disease studies (see Lovell, this issue).

12 The complete quotation from the Holy Bible, King James Version Mark 9:14–29 is as follows: “And when he [Jesus] came to his disciples, he saw a great multitude about them, and the scribes questioning with them. And straightway all the people, when they beheld him, were greatly amazed, and running to *him* saluted him. And he asked the scribes, What question ye with them? And one of the multitude answered and said, Master, I have brought unto thee my son, which hath a dumb spirit; And wheresoever he taketh him, he teareth him: and he foameth, and gnasheth with his teeth, and pineth away: and I spake to thy disciples that they should cast him out; and they could not. He answereth him, and saith, O faithless generation, how long shall I be with you? how long shall I suffer you? bring him unto me. And they brought him unto him: and when he saw him, straightway the spirit tare him; and he fell on the ground, and wallowed foaming. And he asked his father, How long is it ago since this came unto him? And he said, Of a child. And ofttimes it hath cast him into the fire, and into the waters, to destroy him: but if thou canst do any thing, have compassion on us, and help us. Jesus said unto him, If thou canst believe, all things *are* possible to him that believeth. And straightway the father of the child cried out, and said with tears, Lord, I believe; help thou mine unbelief. When Jesus saw that the people came running together, he rebuked the foul spirit, saying unto him, *Thou* dumb and deaf spirit, I charge thee, come out of him, and enter no more into him. And *the spirit* cried, and rent him sore, and came out of him: and he was as one dead; insomuch that many said, He is dead. But Jesus took him by the hand, and lifted him up; and he arose. And when he was come into the house, his disciples asked him privately, Why could not we cast him out? And he said unto them, This kind can come forth by nothing, but by prayer and fasting.”

13 This is particularly pertinent given the fact that many of Ghana’s Pentecostal and Charismatic pastors are self-ascribed and found their own churches and Bible schools. These lack the centralised doctrinal oversight as with the mainstream denominations such as Anglicanism, Methodism and Catholicism, or larger Pentecostal denominations including the international Assemblies of God, and local groups such as the Church of Pentecost (Daswani 2013). Although there is a Ghana Pentecostal and Charismatic Council (GPCC) who have the power to expel members they believe to have contravened doctrine or acted unethically, membership of the council does not seem to influence the popularity of churches, nor does the council seem to have much power over the actions of individual pastors.

14 One pastor who has particularly provoked such debates is the notorious Prophet Obinim (‘Angel Obinim’) who in 2016 was arrested and charged under the Domestic Violence Act after he publicly whipped two adolescent members of his congregation when the girl became pregnant. The event which was video recorded and widely reported in the media provoked fierce debate, with some viewing his actions as justified punishment as the spiritual ‘father’, others seeing them as an infringement of the children’s rights (See https://www.myjoyonline.com/news/2016/August-19th/human-rights-groups-gang-up-against-obinim-demand-his-arrest.php).

15 Such spiritual perception is conveyed by the verb *hu* in Twi which carries the wider meaning of perception as well as to ‘see’. It is not restricted to Christian healers, but also refers to divination as practiced by traditional healers.

16 Health workers expressed more equivocation about working with traditional healers. The use of herbal medicines alongside pharmaceuticals was judged to risk potentially harmful interactions. In addition, many Christian and Muslim health workers view the practices of traditional healers as conflicting with their religious belief.

17 Since this research was conducted many mental health workers have participated in the WHO Quality Rights training which has exposed them to international critiques of involuntary treatment, in particular the rights enshrined in the CRPD. As a consequence some mental health workers are starting to question the routine use of restraint and sedation when treating agitated patients.

18 This has often been cited as the difference between ‘modern’ medicine and traditional healing where the former focuses on the individual and the latter on the social group, as in Turner’s classic description of the Ndembe (Turner 1981). However psychiatric practice of course frequently prioritises the group over the individual, particularly where capacity is deemed to be impaired.

19 Joanna Moncrieff (2013, p. 17) describes how drugs used to treat psychosis have come to be presented as targeting specific disease processes and sedation glossed as a ‘side effect’ rather than a primary action. This glossing of psychotropic drugs as treatment rather than sedation, she argues, enables psychiatrists and other health workers to feel less guilt about the forced drugging of patients. She attributes this to psychiatrists’ desire to distance themselves from the association of sedating pharmaceuticals with psychiatry’s history of mechanical restraint. Though in Ghana psychotropic drugs are also presented by mental health workers as treating a disease, ‘schizophrenia’, they appear less reluctant to promote pharmaceutical sedation as a valuable tool for controlling patient behaviour. Arguments for the government of Ghana to provide a consistent supply of psychopharmaceuticals are often explicitly framed by mental health workers as needed to prevent an outbreak of violence by persons with mental illness.
